# Targeted accumulation of selective anticancer depsipeptides by reconstructing the precursor supply in the neoantimycin biosynthetic pathway

**DOI:** 10.1186/s40643-021-00397-z

**Published:** 2021-05-22

**Authors:** Lin Zhou, Yaoyao Shen, Nannan Chen, Wanlu Li, Hou-wen Lin, Yongjun Zhou

**Affiliations:** Research Center for Marine Drugs, State Key Laboratory of Oncogenes and Related Genes, Department of Pharmacy, Ren Ji Hospital, School of Medicine, Shanghai Jiao Tong University, Shanghai, 200127 People’s Republic of China

**Keywords:** Anticancer, Depsipeptides, Natural product biosynthesis, 3-Hydroxybenzoate, 3-Formamidosalicylate, Chorismic acid

## Abstract

**Background:**

Neoantimycins are a group of 15-membered ring depsipeptides isolated from *Streptomycetes* with a broad-spectrum of anticancer activities. Neoantimycin biosynthesis is directed by the hybrid multimodular megaenzymes of non-ribosomal peptide synthetase and polyketide synthase. We previously discovered a new neoantimycin analogue unantimycin B, which was demonstrated to have selective anticancer activities and was produced from the neoantimycin biosynthetic pathway with a starter unit of 3-hydroxybenzoate, instead of the 3-formamidosalicylate unit that is common for neoantimycins. However, the low fermentation titre and tough isolation procedure have hindered in-depth pharmacological investigation of unantimycin B as an anticancer agent.

**Results:**

In this work, we genetically constructed two unantimycin B producer strains and inhibited neoantimycins production by removing *nat*O *and nat*J-L genes essential for 3-formamidosalicylate biosynthesis, therefore facilitating chromatographic separation of unantimycin B from the complex fermentation extract. Based on the Δ*nat*O mutant, we improved unantimycin B production twofold, reaching approximately 12.8 mg/L, by feeding 3-hydroxybenzoate during fermentation. Furthermore, the production was improved more than sixfold, reaching approximately 40.0 mg/L, in the Δ*nat*O strain introduced with a chorismatase gene highly expressed under a strong promoter for endogenously over-producing 3-hydroxybenzoate.

**Conclusion:**

This work provides a case of targeting accumulation and significant production improvement of medicinally interesting natural products via genetic manipulation of precursor biosynthesis in *Streptomycetes*, the talented producers of pharmaceutical molecules.

**Figure:**
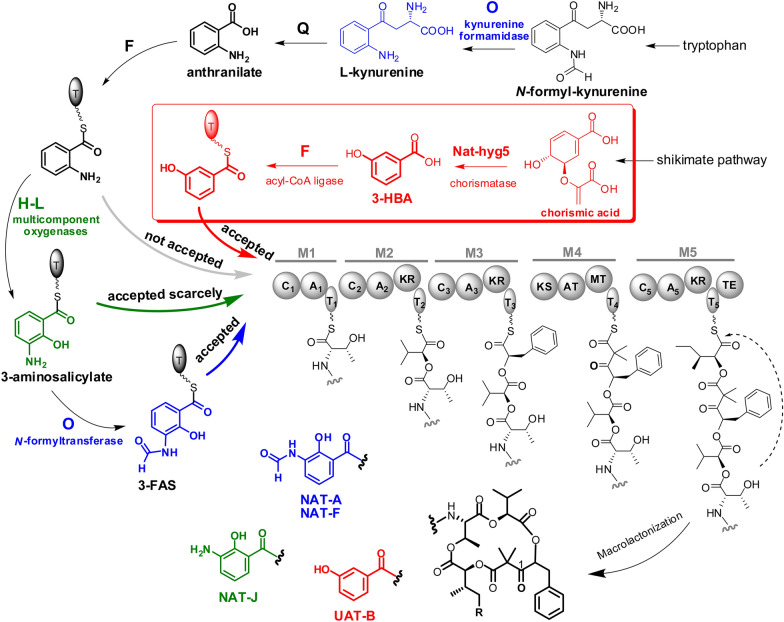

**Supplementary Information:**

The online version contains supplementary material available at 10.1186/s40643-021-00397-z.

## Introduction

**Fig. 1:**
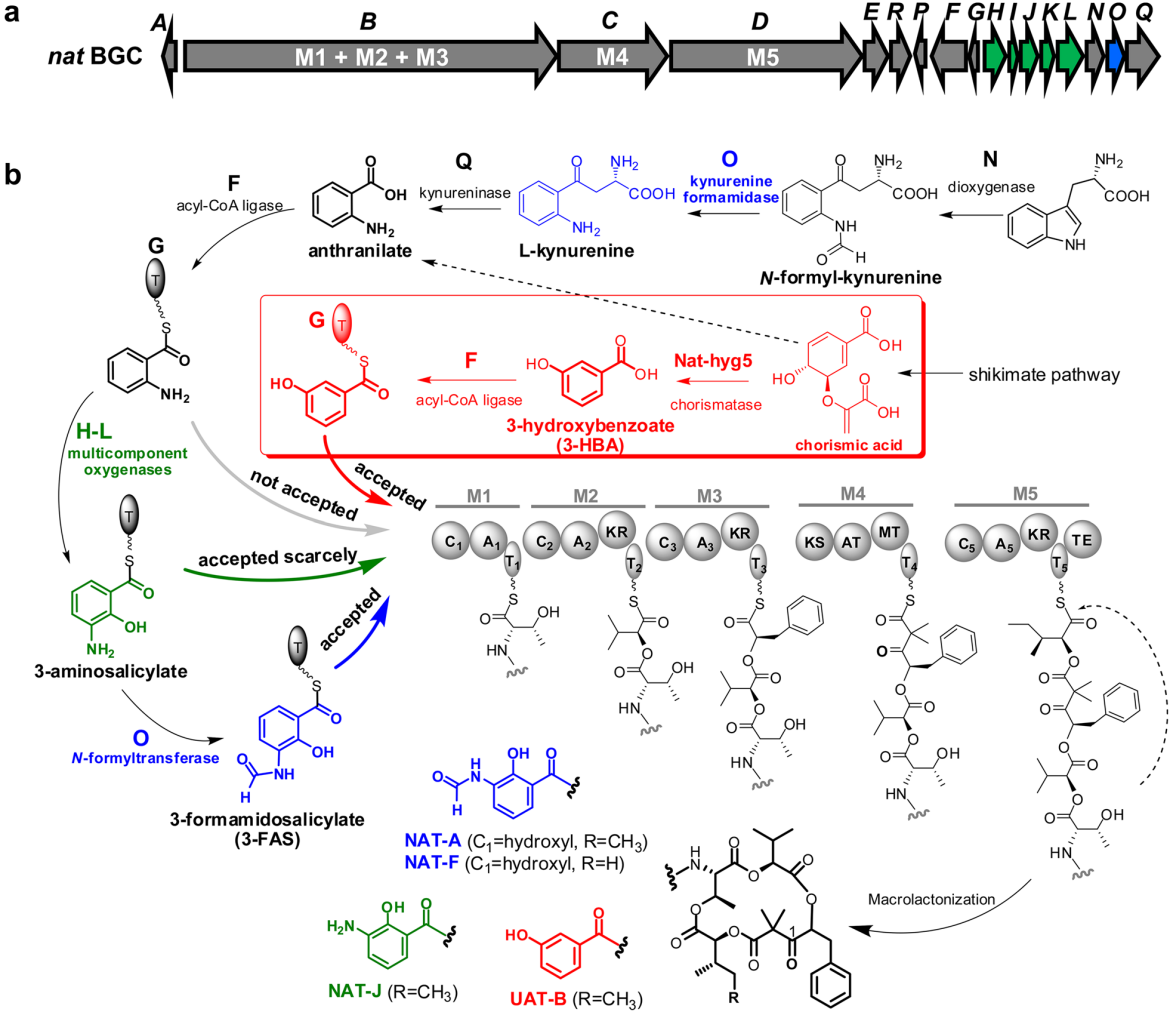
The gene organization of the NAT biosynthetic gene cluster (*nat* BGC) (**a**) and the biosynthetic routes of the starting precursor for the NAT NRPS-PKS assembly line (**b**). The genes and related products of *nat*H-L and *nat*O are marked in green and blue, respectively. The bio-reaction schemes of 3-hydroxybenzoate (3-HBA) generation and loading processes are highlighted in red. The dashed arrow represents that anthranilate could be produced from chorismic acid via primary aromatic amino acid metabolism. A: adenylation; T: thiolation or acyl-carrier protein; C: condensation; KR: ketoreductase; KS: ketosynthase; AT: acyltransferase; MT: methyltransferase; TE: thioesterase

*Streptomyces* species are a family of Gram-positive bacteria renowned for their ability to produce a multitude of secondary metabolites with pharmaceutical activities (Van der Heul et al. 2018). Neoantimycins (NATs) are anticancer compounds of 15-membered ring antimycin-type depsipeptides discovered from the fermentation extract of *Streptomycetes* (Liu et al. 2016, 2019; Li et al. 2013). The molecular skeleton of NATs was biosynthetically assembled by a hybrid multimodular protein complex of non-ribosomal peptide synthetase (NRPS) and polyketide synthase (PKS), with the starting precursor of 3-formamidosalicylate (3-FAS) (Zhou et al. 2018; Skyrud et al. 2018). We previously discovered a new NAT derivative, unantimycin B (UAT-B), from the fermentation extract of terrestrial *Streptomyces conglobatus* and verified that the biosynthesis of UAT-B was directed by the NAT NRPS-PKS with a starter unit of 3-hydroxybenzoate (3-HBA) (Shen et al. 2020) (Fig. 1). UAT-B was demonstrated to process remarkable inhibitory activities against human lung cancer, colorectal cancer, and melanoma cells with no cytotoxic activities towards the corresponding noncancerous cells (Shen et al. 2020). However, UAT-B was produced as a minor product at a yield of 1 to 2% of the main product (NAT-A) in the wild-type strain. Although the yield level of UAT-B increased relative to that of NAT-A in the heterologous expression host of *S. albus* J1074 (Shen et al. 2020), the production titre of UAT-B (approximately 6.3 mg/L) remained at the same low level as that of the wild-type strain (Additional file 1: Fig. S1). Moreover, the presence of the main product NAT-A seriously interrupted the chromatographic separation of UAT-B. Owing to these challenges, UAT-B had to be prepared with a challenging isolation procedure from the complex fermentation extract, therefore hindering in-depth pharmacological investigation of the compound as an anticancer agent.

Inspired by the documented approaches of increasing biosynthetic precursor supply and manipulating the substrate specificity of the key enzyme to improve the yields or purity of target metabolites in bacteria (Liu et al. 2018; Fries et al. 2019), we sought to address the above difficulties in this work. Herein, we show the work of successfully directing the NAT NRPS-PKS towards UAT-B production from the original pathway for NATs production by genetically blocking 3-FAS biosynthesis. Based on the resultant mutant with a relatively clear fermentation background, we further achieved significant UAT-B production improvement by enhancing the supply of the starter unit precursor via both exogenous and endogenous strategies. In addition, we obtained some insight for further UAT-B production optimization and investigated the starter unit tolerance of the NAT NRPS-PKS. This work displays a case of targeting accumulation and significant production improvement of medicinally interesting compounds through genetically manipulating precursor supply in the natural product biosynthetic pathway from *Streptomycetes*, the genius bacterium in producing diverse pharmaceutical molecules.

## Materials and methods

### DNA manipulation and chemicals

The oligonucleotides used in this work were synthesized by Shanghai Generay Biotech Co., Ltd. Restriction endonucleases and T4 DNA ligase were purchased from New England Biolabs. DNA fragment assembly was performed by using the U-Clone master mix kit (Evomic Science, Sunnyvale, CA, USA). Chemicals were purchased from Sigma-Aldrich. Plasmid DNA extraction was performed using a plasmid mini kit (Shanghai Generay Biotech). Genomic DNA used as a PCR template was prepared by using 10% Chelex 100 resin (Bio-Rad) solution. PCR amplifications were carried out by using Phusion High-Fidelity PCR Master Mix (New England Biolabs) for DNA cloning or 2× FastTaq Master Mix (Shanghai Bioroot Biotech) for colony screening.

### Strains, media, and culture conditions

RJ2 was derived from the terrestrial *S. conglobatus* and used as a starting producer of UAT-B (Shen et al. 2020). *E. coli* DH10B was used for plasmid construction. *E. coli* ET12567 containing the helper plasmid of pUZ8002 was used as a transitional host for introducing the target plasmid into RJ2. TSBY medium (3% tryptone soy broth, 0.5% yeast extract, 10% sucrose, 0.1% antifoam) was used to produce mycelium of RJ2. SFM agar medium (2% soya flour, 2% d-mannitol, 2% agar) was used for manipulation of conjugation and mutant screening for RJ2. Fermentation production medium (SGC) consisting of 3% soybean flour, 5% glucose, 0.5% CaCO_3_, and 0.1% (v/v) antifoam was used to produce UAT-B. *Streptomyces* fermentation was carried out in a 250-mL conical flask fitted with a metal spring, with 1% (v/v) inoculation of a 3-day TSBY culture into 50 mL of SGC medium, and then incubated at 30 °C and 220 rpm for 5 days. *E. coli* strains were grown in Luria–Bertani (LB) broth (1% tryptone, 0.5% yeast extract, 0.5% NaCl) or LB agar (1.5% agar) at 37 °C with the corresponding antibiotics supplemented.

### Metabolite extraction and HPLC–MS analysis

To extract fermentation metabolites for HPLC–MS analysis, 7 mL of 5-day SGC fermentation broth was extracted with an equal volume of ethyl acetate. The extract was dried by vacuum evaporation and re-dissolved in 500 μL of methanol. Twenty microlitres of the final sample was injected for HPLC–MS analysis.

HPLC–MS analysis was conducted on a Waters HPLC coupled with a Waters Acquity QDa detector. The analytical HPLC instrument was fitted with a Waters Xbridge C18 column (250 mm × 4.6 mm, 5 μm). Samples were eluted with the mobile phases of acetonitrile and aqueous 0.1% formic acid (v/v) at a flow rate of 0.7 mL/min with 80% of acetonitrile over 30 min or with a gradient elution of 30–100% acetonitrile over 30 min. The mass spectrometer was run in positive ionization mode, scanning from *m*/*z* 200 to 1250.

### Quantitative analysis of UAT-B production levels

Quantitative analysis of UAT-B production titres from the fermentation extracts was conducted by using an Agilent 1290 Infinity ultra performance liquid chromatography (UPLC) instrument and an Agilent 6460 triple quadrupole mass spectrometer (QqQ/MS). The UPLC was fitted with a Waters ACQUITY UPLC HSS T3 column (50 mm × 2.1 mm, 1.8 μm) at 35 °C. A solvent system of acetonitrile and aqueous 0.1% formic acid (v/v) was used for gradient elution with acetonitrile at 60 to 95% over 4 min and 95% for 1 min at a flow rate of 0.3 mL/min. The mass spectrometer was run in positive ionization mode with a gas temp 315 °C, a nebulizer pressure of 45 psi, a sheath gas temperature of 300 °C, a capillary voltage of 4 kV, and a nozzle voltage of 500 V. To produce a standard curve for quantitation, a stock DMSO solution of UAT-B (653 mg/L) was diluted 10, 20, 50, 100, 500, and 1000 times by using a methanol solution of the fermentation extract from RJ15, a RJ2 derived mutant not exhibiting UAT-B production (Shen et al. 2020). The original data were collected and calculated by Agilent MS workstation software B.08.00. The standard curve was subjected to linear regression with 1/X weighting. All the samples were analysed in triplicate.

### Fermentation feeding

DMSO solutions of 3-HBA and l-kynurenine were prepared at concentrations of 1.0 M and 0.5 M, respectively. Feeding was carried out by adding 50 μL of 3-HBA or 100 μL of l-kynurenine to 50 mL of SGC medium on the 2nd and 4th days of fermentation, respectively. The broth was extracted for analysis after 5 days of fermentation.

### Deletion of the *nat*O gene

To construct two homologous recombination arms for in-frame deletion of *nat*O, the primers L-O-S and L-O-A and primers R-O-S and R-O-A (Table 1) were used to amplify a 1499 bp left arm and a 1381 bp right arm, respectively. The two PCR fragments were assembled together by using U-Clone master mix kit with the *E. coli*–*Streptomyces* shuttle plasmid pRJ2 linearized with *Xba*I and *Eco*RI (Zhou et al. 2018). The resulting plasmid pRJ65 (Table 2) was transformed into RJ2 via conjugation from a transitional host of *E. coli* ET12567 with the helper plasmid pUZ8002. The target mutant was screened with the primers of C-O-S and C-O-A (Table 1) from the hygromycin-sensitive colonies prepared after two rounds of propagation on SFM plates without antibiotic addition. The expected PCR products were 1021 bp for the target mutation and 1507 bp for RJ2. The PCR products were sequenced to finally confirm the target mutant ∆*nat*O.

**Table 1:** PCR primers used in the study

Primer name	Sequence (5′-3′)
L-O-S	ATCCCCGGGGACCTGCAGGTCGACTACGACGCCCACTTCTACCTCT
L-O-A	ATAGCCGGTGAAGTAGCCGCCGTGGAGCAGCACCACCAACG
R-O-S	ACGGCGGCTACTTCACCGGCTATCGGGCCGTGCACAAG
R-O-A	TATCACGAGGCCCTTTCGTCTTCAAGAGACGTAGAGCGCGTTGACGCC
C-O-S	TCTACGACGAGGTGCTGGGCTTTCTGC
C-O-A	AAGAGCTGGACGCTGGTGGAATCGC
L-JL- S	ATCCCCGGGGACCTGCAGGTCGACTACGACGTGCTGTGGTCGCTG
L-JL- A	AGAGGTAGAAGTGGGCGTCGTCCAGCTCGTCGCTCTCGAACAGCTC
R-JL- S	TTCGAGAGCGACGAGCTGGACGACGCCCACTTCTACCTCTG
R-JL- A	TATCACGAGGCCCTTTCGTCTTCAAGTGGAGCAGCACCACCAAC
JL-T-S	AGTTCCGCAACGTCCTCCTG
JL-T-A	AGAACAGCTCGAAACGCACC
phiC31-F	TGAGCTCATGAGCGGAGAACGAG
phiC31-R	TTTTACAAACTTCTCGACAGACGTAGATCAG
puc-F	TACGTCTGTCGAGAAGTTTGTAAAACGACGGCCAGTGCCA
puc-R	AGGCGACGGTGTACGCCATATTGATGACATCAGTCGATC
hyg-F	ATCGTGCTATGATCGACTGATGTCATCAATATGGCGTACACCGTCGCCTC
hyg-R	ATCTCGTTCTCCGCTCATGAGCTCAGGCGCCGGGGGCGGT
ermE-pRJ71-S	AAGCTTGGGCTGCAGGTCGACTCTAGTATGCATGCGAGTGTC
hyg5-pRJ5-A	AAACAGCTATGACATGATTACGAATTCGATATCAGGCGATGATGCCCTC
kasO-hyg5-S	ACAGCGTGCAGGACTGGGGGAGTTATGCTGCGATGCGATTAC
SP-hyg5-S	AGAGACAGACCCCCGGAGGTAACCCATATGCTGCGATGCGATTAC
pRJ5-S2	AAGACGTAGCGGCGTAGCGAGAC
pIB139-A	TGAGTTAGCTCACTCATTAGGCAC
AntF-S	TGCGGACCATCGCCGACTACGAGCGGTTCGTC
AntF-A	AGCTATGACATGATTACGAATTCGATATCAGGCGCGCAGCGCCTTCTTC
NatF-S	GTGCCTGATGTGGCTGACCCAGTTGCTGGAGCGCAAC
NatF-A	TGTTGTGTGGAATTGTGAGCGGATAACAATTTCACACAGGAAACAG
AACL-A	ATCAGGTCCGCAGCAGTTTCTTGAG

**Table 2:** Bacterial strains and plasmids used in the study

Strains/plasmids	Application characteristics and antibiotic resistances	Sources/references
pRJ2	*E. coli*–*Streptomyces* shuttle plasmid used for constructing gene deletion, hygR, blaR	Zhou et al. (2018)
pRJ5	pIB139 derivative containing *ermE*p* and *phiC*31 attP site, hygR	This work
pRJ65	pRJ2 derivative used for in-frame deletion of *nat*O, hygR, blaR	This work
pRJ138	pRJ2 derivative used for deletion of *nat*J-L, hygR, blaR	This work
pRJ71	PCR template used to amplify *ermE*p*_*nat*-hyg5 cassette, apramycin and thiostrepton resistances	Shen et al. (2020)
pRJ251	*ermE*p*_*nat*-hyg5 cassette in pRJ5 (NsiI, EcoRV), hygR, *phiC*31 attP site	This work
pRJ252	*kasO*p*_*nat*-hyg5 cassette in pRJ5 (NsiI, EcoRV), hygR, *phiC*31 attP site	This work
pRJ253	*A4*p_*nat*-hyg5 cassette in pRJ5 (NsiI, EcoRV), hygR, *phiC*31 attP site	This work
pRJ255	*kasO*p*_antG*-nat*F + *A4*p_*nat*-hyg5 in pRJ5 (NsiI, EcoRV), hygR, *phiC*31 attP site	This work
pRJ256	*kasO*p*_antGF + *A4*p_*nat*-hyg5 in pRJ5 (NsiI, EcoRV), hygR, *phiC*31 attP site	This work
RJ2	The mutant of *S. conglobatus* losing conglobatin production	Shen et al. (2020)
∆*nat*O	In-frame deletion of *nat*O gene from RJ2	This work
∆*nat*J-L	Deletion of *nat*J-L genes from RJ2	This work

*hygR*, hygromycin resistance, *blaR*, ampicillin resistance

### Deletion of the *nat*J-L genes

To generate a deletion within *nat*J-L genes of the *nat*H-L operon (Fig. 1a), homologous recombination arms were produced by using the primers L-JL-S and L-JL-A for the 1378-bp left arm and the primers R-JL-S and R-JL-A for the 1497-bp right arm (Table 1). The two PCR products were assembled together in pRJ2 (*Xba*I and *Eco*RI) by using the same strategy as described above. The resulting plasmid pRJ138 (Table 2) was transformed into RJ2 via conjugation. The target mutant was screened with the primers of JL-T-S and JL-T-A (Table 1) from the hygromycin-sensitive colonies prepared after two rounds of propagation on SFM plates without antibiotic addition. The expected PCR products were 408 bp for the target mutation and 1782 bp for the parent strain RJ2. The PCR products were finally sequenced to confirm the target mutant ∆*nat*J-L.

### Construction of *nat*-hyg5 gene expression cassettes

The open reading frame (ORF) of the *nat*-hyg5 gene combined with different promoters was introduced into the ∆*nat*O strain via an integrative vector pRJ5 containing phage *phiC*31 integration elements (Table 2). pRJ5 was constructed by using a U-Clone master mix kit from three fragments, including the two fragments amplified from pIB139 (Wilkinson et al. 2002) with the primers phiC31-F and phiC31-R for 3241 bp and the primers puc-F and puc-R for 1536 bp, and the 1239 bp fragment of the hygromycin resistance gene amplified with the primers hyg-F and hyg-R from pMS82 (Gregory et al. 2003) (Table 1). The *nat-*hyg5 gene expression cassettes were constructed with the promoters *ermE*p* (Wilkinson et al. 2002), *kasO*p* (Wang et al. 2013), and *A4*p (Fox and Wang 2008) based on pRJ5 (*Nsi*I, *EcoR*V) to produce the plasmids pRJ251, pRJ252, and pRJ253, respectively (Table 2). The fragments of *kasO*p* and *A4*p were ordered from Union-Biotech (Shanghai) Co., Ltd. pRJ251 was generated by assembling pRJ5 (*Nsi*I, *EcoR*V) and the 1243 bp fragment amplified from pRJ71 (Shen et al. 2020) with the primers ermE-pRJ71-S and hyg5-pRJ5-A (Tables 1 and 2). pRJ252 was assembled from the vector pRJ5 (*Nsi*I, *EcoR*V) and the two inserts of 214 bp *kasO*p*, and the 1051 bp fragment amplified from pRJ71 with the primers kasO-hyg5-S and hyg5-pRJ5-A (Tables 1 and 2). pRJ253 was assembled from the vector pRJ5 (*Nsi*I, *EcoR*V) and the two inserts of 492 bp *A4*p, and the 1054-bp fragment amplified from pRJ71 with the primers SP-hyg5-S and hyg5-pRJ5-A (Tables 1 and 2). The resultant plasmids were transformed into ∆*nat*O via conjugation, respectively. The exconjugants were confirmed via the phenotype of hygromycin resistance and colony PCR with the primers pRJ5-S2 and pIB139-A (Table 1).

### Construction of *ant*G-*nat*F and *ant*GF gene expression cassettes

The gene expression cassettes of *ant*G*-nat*F and *ant*G-*ant*F (*ant*GF) under the promoter of *kasO*p* were introduced into the *Nsi*I site of pRJ253 to give plasmids pRJ255 and pRJ256, respectively (Table 2). The two cassettes were generated by assembling a 426-bp synthesized *kasO*p*_*ant*G fragment with the 1585-bp *ant*F and the 1566-bp *nat*F fragments, respectively. The 1585-bp *ant*F was amplified with the primers AntF-S and AntF-A from *S. albus* J1074 (Yan et al. 2012). The 1566-bp *nat*F was amplified with the primers NatF-S and NatF-A from RJ2. The resulting plasmids were transformed into ∆*nat*O via conjugation. The exconjugants were confirmed by observing hygromycin resistance and colony PCR with the primers pRJ5-S2 and AACL-A (Table 1).

## Results and discussion

### Generation of UAT-B producers with blocked NATs production

**Fig. 2:**
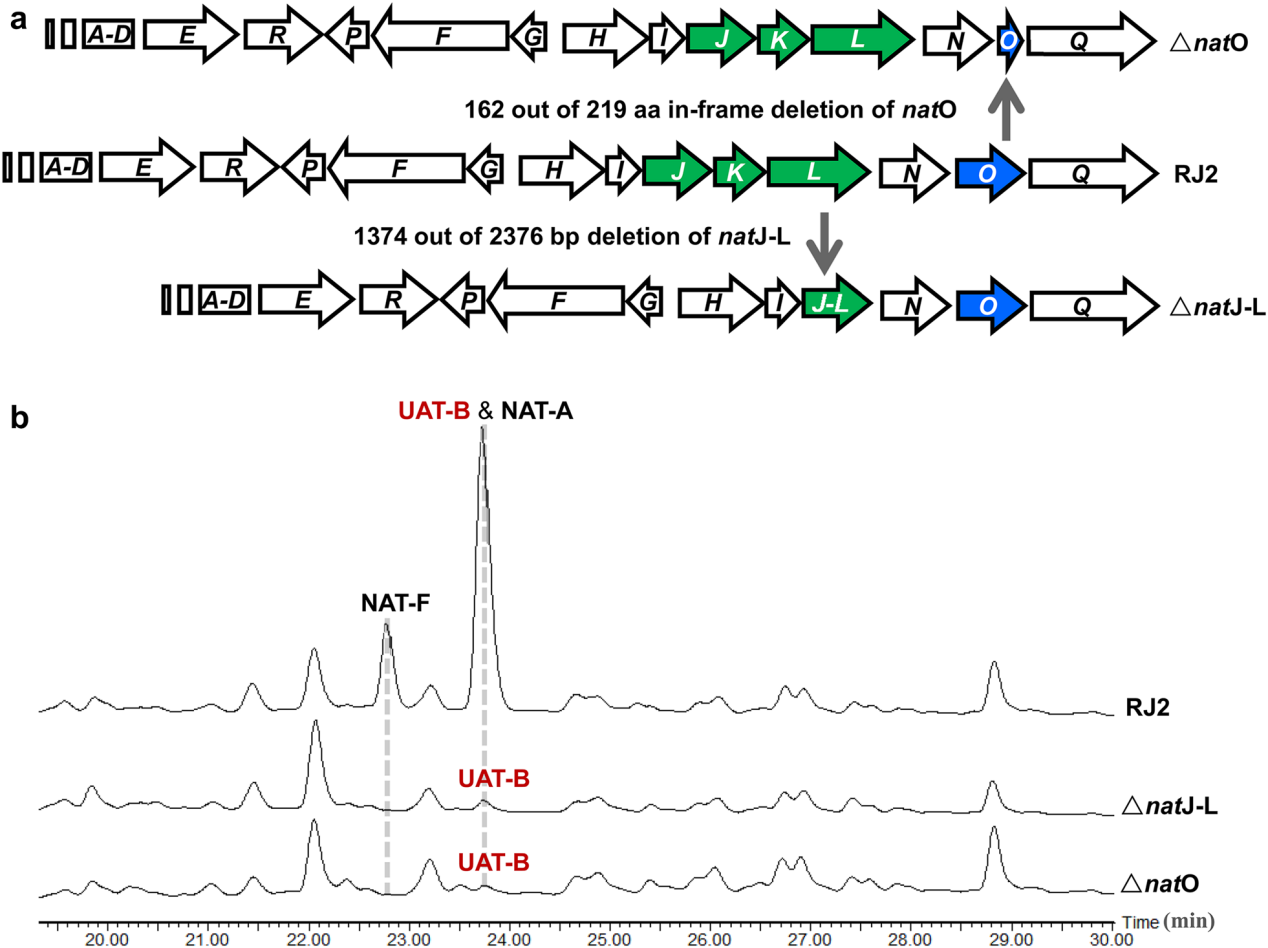
Schematic diagrams of generating Δ*nat*O and Δ*nat*J-L from RJ2 (**a**). HPLC analysis of UAT-B, NAT-A and NAT-F from the fermentation extracts of the parent strain RJ2 and the mutants Δ*nat*O and Δ*nat*J-L (**b**). The data is displayed with UV320 extraction

To generate a strain with blocked NAT production that exclusively produced UAT-B, the biosynthesis of 3-FAS was blocked via gene deletion of *nat*O putatively involved in both the *N*-deformylation of *N*-formyl-kynurenine and the *N*-formylation of 3-aminosalicylate or the *nat*H-L genes putatively responsible for epoxidation and a 1,2-shift of the thioester group in 3-FAS biosynthesis (Fig. 1b). To create these mutations, the two *E. coli–Streptomyces* shuttle plasmids, pRJ65 containing an in-frame deletion (162 out of 219 aa) in *nat*O and pRJ138 containing a deletion (1374 out of 2376 bp) in the *nat*J-L gene operon were constructed (Table 2). The expected mutations in *nat*O and *nat*J-L genes were then introduced into the chromosome of the parent strain RJ2 (Shen et al. 2020) through homologous recombination (Fig. 2a). As expected, NAT-A and NAT-F disappeared in Δ*nat*O and Δ*nat*J-L mutant strains and UAT-B production remained in both mutants according to HPLC–MS analysis of the fermentation extract (Fig. 2b). We originally proposed that UAT-B titre would be higher than that from the parent strain since NAT NRPS-PKS was exclusively utilized to synthesize UAT-B when 3-HBA was unavailable in the mutants. However, the expected increase in UAT-B production was not observed in the Δ*nat*O or Δ*nat*J-L mutant compared to that in RJ2. The phenotype suggested that insufficient supply of 3-HBA could be a bottleneck for UAT-B production, and supplementation with 3-HBA in fermentation should increase UAT-B production.

### Improvement in UAT-B production by feeding 3-HBA in fermentation

**Fig. 3:**
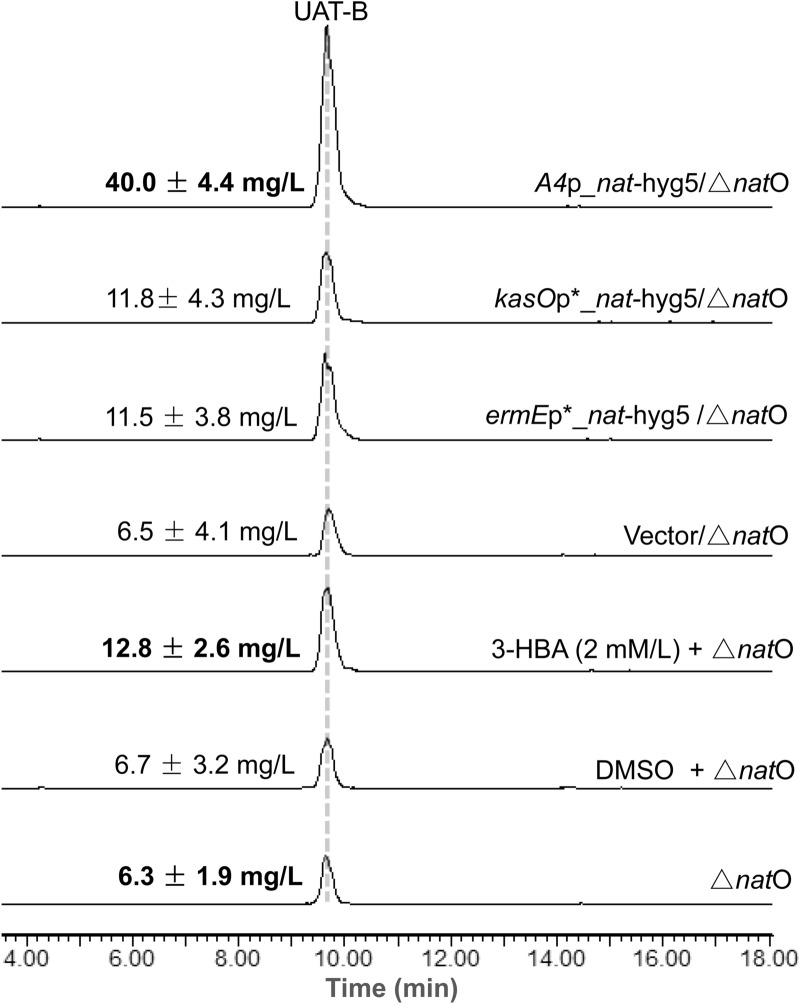
HPLC–MS analysis of UAT-B production in the fermentation extracts of the Δ*nat*O strain fed 3-HBA or containing different *nat*-hyg3 gene expression cassettes: *A4*p*_nat-*hyg5, *kasO*p*_*nat-*hyg5 or *ermE*p*_*nat-*hyg5. The HPLC–MS data are displayed with the mass extraction of [M+Na]^+^ *m*/*z* 676.4 for UAT-B. The exact UAT-B production titres displayed were obtained by using UPLC-QqQ/MS analysis. All the production titre values shown are the mean ± standard deviation of triplicate determinations

To evaluate whether the 3-HBA supply was a rate-limiting step in improving UAT-B production, a DMSO solution of 3-HBA (1 M) was added twice in a liquid fermentation of the Δ*nat*O strain to a final concentration of 2 mM. HPLC–MS analysis of the resulting fermentation extract indicated that UAT-B production was notably improved compared to the control supplemented with DMSO (Fig. 3). Further quantitative analysis by using UPLC-QqQ/MS confirmed the production titre of UAT-B as 12.8 ± 2.6 mg/L with 3-HBA feeding, approximately twice the titre (6.3 ± 1.9 mg/L) from fermentation without 3-HBA feeding. The results therefore confirmed our hypothesis that increasing the 3-HBA supply would increase UAT-B production.

### Improvement in UAT-B production by overexpressing the gene involved in 3-HBA biosynthesis

To generate a high UAT-B-producing strain independent of the exogenous supply of 3-HBA, the Δ*nat*O strain was used as a host to overexpress the *nat*-hyg5 gene, which was verified to encode a chorismatase capable of converting endogenous chorismate into 3-HBA in RJ2 (Shen et al. 2020) (Fig. 1b). It is worth mentioning that the Δ*nat*J-L strain was not chosen as a host because anthranilate could compete with 3-HBA in the process of being loaded onto an acyl-carrier protein (NatG) by NatF (Fig. 1b). Moreover, considering that heterologous promoters could perform differently in a new host or fermentation medium (Wang et al. 2013), three strong and constitutive promoters were studied, including *ermE*p* (Wilkinson et al. 2002), *kasO*p* (Wang et al. 2013), and *A4*p (Fox and Wang, 2008). Therefore, the gene expression cassettes of *ermE*p*_*nat-*hyg5, *kasO*p*_*nat-*hyg5, and *A4*p_*nat-*hyg5 were constructed based on the phiC31 integrative vector pRJ5. The resulted plasmids pRJ251, pRJ252, and pRJ253 (Table 2) were conjugated into the Δ*nat*O strain, respectively. According to HPLC–MS analysis, significant UAT-B production improvement was obtained upon each of the three *nat-*hyg5 cassettes compared to the empty vector control (Fig. 3). Among the three cassettes, *A4*p_*nat-*hyg5 gave the highest production of UAT-B as determined by using UPLC-QqQ/MS analysis, giving a titre of 40.0 ± 4.4 mg/L, more than three times that achieved by feeding 3-HBA and more than six times the titre of the starting Δ*nat*O strain, 6.3 ± 1.9 mg/L (Fig. 3).

### Investigation of UAT-B production by overexpressing the genes involved in loading the starter unit

Considering another possibility that UAT-B production could be limited at the stage of loading the starting precursor, in which acyl-CoA ligase (NatF) authentically loaded anthranilate, rather than 3-HBA, onto the acyl-carrier protein (NatG) in the 3-FAS biosynthetic pathway (Fig. 1b), we decided to further optimize UAT-B production by raising the expression levels of NatG and NatF based on the host of the Δ*nat*O strain. As documented, *nat*GF genes could be functionally replaced by the homologous genes of *ant*GF, which were derived from the antimycin biosynthetic gene cluster and showed 80–90% identity to *nat*GF in the protein sequence (Zhou et al. 2018). Moreover, AntF (80% identity to NatF) was demonstrated to have a wide range of substrate specificities (Yan et al. 2012; Sandy et al. 2012), encouraging us to investigate whether AntF was more tolerant than NatF in loading 3-HBA onto the acyl-carrier protein (AntG). We therefore designed two plasmids to co-express the *nat-*hyg5 gene with the *ant*G*-nat*F and *ant*G-*ant*F (*ant*GF) cassettes, respectively, to compare the contributions of *nat*F and *ant*F to UAT-B production improvement. Finally, the cassettes of *kasO*p*_*ant*G-*nat*F and *kasO*p*_*ant*GF were introduced into the plasmid pRJ253 containing *A4*p_*nat*-hyg5 to generate pRJ255 and pRJ256, respectively. However, neither of the cassettes increased UAT-B production in the Δ*nat*O strain, as determined by HPLC–MS analysis (Additional file 1: Fig. S2). We hypothesized that an alternative rate-limiting step could be the first condensation stage of NAT NRPS-PKS, in which the condensation domain (C_1_) formed an amide bound between the starter unit, e.g., 3-HBA or 3-FAS, tethered on NatG and the threonine loaded on the thiolation domain (T1). It was therefore possible to further improve UAT-B production by increasing the preference of C_1_ towards 3-HBA. Moreover, tuning the substrate specificity of NatF (acyl-CoA ligase) towards 3-HBA through site-mutation could be another candidate strategy.

### Investigation of the starter unit tolerance of NAT NRPS-PKS by analysing the metabolites from Δ*nat*O and Δ*nat*J-L mutants

**Fig. 4:**
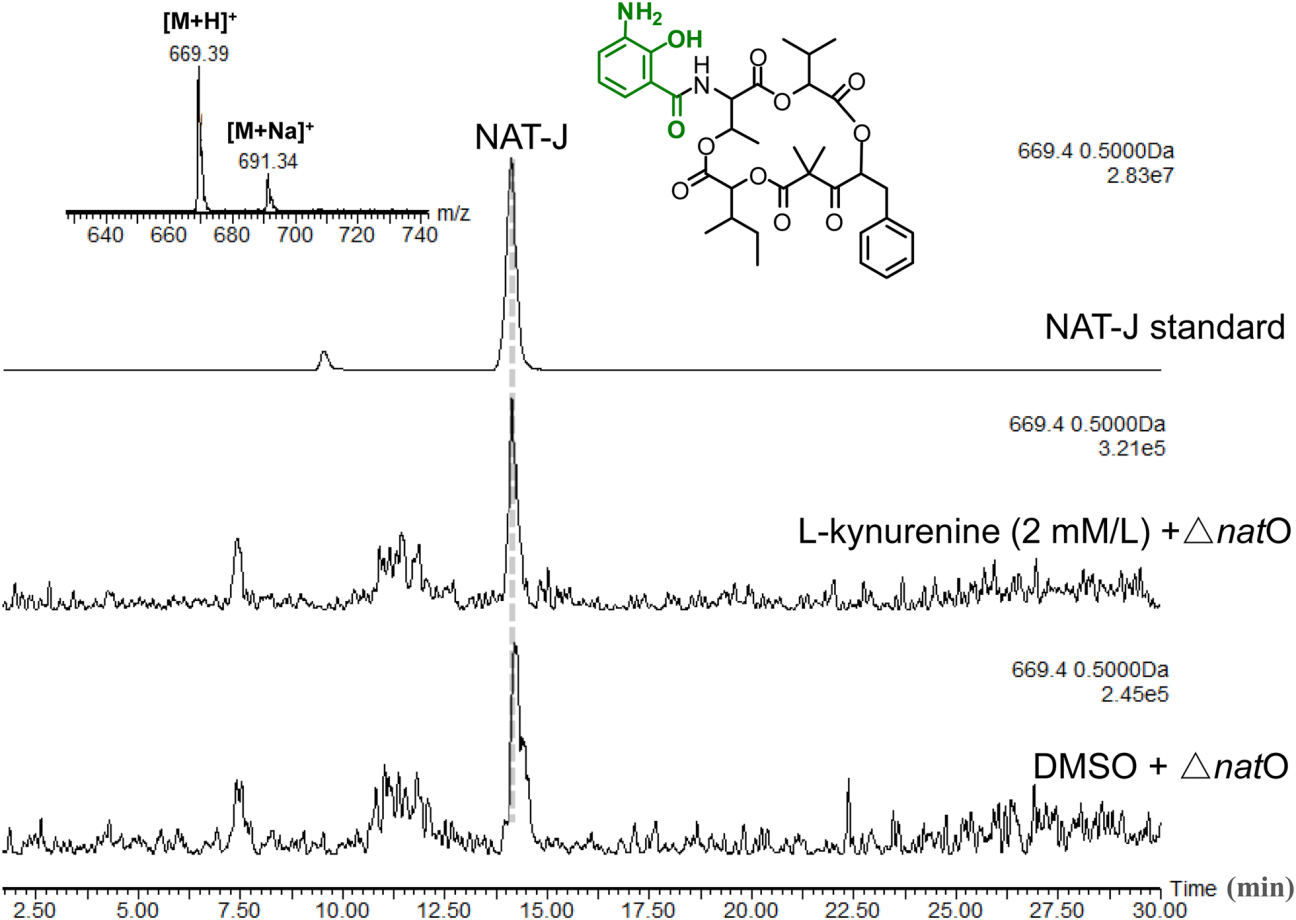
HPLC–MS analysis of NAT-J production in the fermentation extracts of the Δ*nat*O strain fed l-kynurenine (2 mM) or DMSO. HPLC–MS data are displayed with the mass extraction of [M+H]^+^ *m*/*z* 669.4 for NAT-J

The structural features of reported NAT analogues suggest that NAT NRPS-PKS can accept diverse starting precursors, such as benzoic acid, 3-HBA, and 3-aminosalicylic acid, except 3-FAS (Izumikawa et al. 2007; Lim et al. 2016; Lin et al. 2019). Taking advantage of the Δ*nat*J-L mutant generated in the work, we explored whether NAT NRPS-PKS could accept anthranilate as a starter unit. According to the proposed 3-FAS biosynthesis, *nat*H-L encodes the multicomponent oxygenase catalysing epoxidation and a 1,2-shift of the thioester group in anthraniloyl-*S*-NatG to produce 3-aminosalicyloyl-*S*-NatG (highlighted in green, Fig. 1b) (Schoenian et al. 2012; Liu et al. 2015); thus, disruption of *nat*H-L would accumulate anthranilate as a starter unit to produce new NAT analogues. However, no expected products were detected in the fermentation extract of Δ*nat*J-L by using HPLC–MS, suggesting that anthranilate did not result in a product in NAT NRPS-PKS. Moreover, by using the *nat*O mutant, we evaluated whether 3-aminosalicylate could be more efficiently loaded as a starter unit to produce NAT-J, a minor product putatively derived from the NAT biosynthetic pathway (Lin et al. 2019). In the proposed 3-FAS biosynthesis, NatO, showing 76% identity to the AntO derived from the antimycin biosynthetic pathway, was believed to work as a difunctional enzyme conducting two catalytic processes: the deformylation of *N*-formyl-kynurenine to generate l-kynurenine and the *N*-formylation of 3-aminosalicylate to produce 3-FAS (highlighted in blue, Fig. 1b) (Liu et al. 2015; Zhou et al. 2018). Thus, Δ*nat*O should lose NAT-J production due to the destroyed supply of l-kynurenine. However, NAT-J production in Δ*nat*O was still observed at a similar level as that in the parent strain, suggesting that the blocked l-kynurenine could be complemented, for instance, by the anthranilate produced from primary aromatic amino acid metabolism (Sprenger 2007) (Fig. 1b). Moreover, because NatQ, NatF, NatG, and NatH-L worked efficiently in NAT-A biosynthesis, we hypothesized that exogenously supplemented l-kynurenine could be processed sufficiently to deliver 3-aminosalicylate for NAT-J production (Fig. 1b). However, supplementation with 2 mM l-kynurenine did not increase NAT-J production in the Δ*nat*O strain according to HPLC–MS analysis (Fig. 4), indicating that 3-aminosalicylate could only be incorporated as a starter unit by NAT NRPS-PKS to a very low extent.

## Conclusions

NATs are a group of 15-membered ring depsipeptides with a broad spectrum of anticancer activities (Liu et al. 2016, 2019). NAT biosynthesis is directed by a hybrid multimodular protein complex of non-ribosomal peptide synthetase (NRPS) and polyketide synthase (PKS) with the starting precursor of 3-formamidosalicylate (3-FAS) (Zhou et al. 2018; Skyrud et al. 2018). We previously discovered the new NAT derivative unantimycin B (UAT-B), which exhibited selective anticancer activities and was verified to be produced by NAT NRPS-PKS with a starter unit of 3-hydroxybenzoate (3-HBA) rather than 3-FAS (Shen et al. 2020) (Fig. 1b). However, UAT-B was produced in low yield (approximately 6.3 ± 1.9 mg/L), and it was difficult to chromatographically separate UAT-B from the dominant product NAT-A in the complex fermentation extract (Fig. 2b), therefore hindering in-depth pharmacological investigation of the compound as an anticancer agent. In this work we blocked NAT-A and NAT-F production via deletion of *nat*O or *nat*J-L genes essential to 3-FAS biosynthesis to direct NAT NRPS-PKS to exclusively produce UAT-B (Figs. 1 and 2). However, the expected production improvement in UAT-B was not observed in the mutants, implying that insufficient supply of 3-HBA could be a bottleneck for improving UAT-B production. This proposal was then supported by the results that feeding 3-HBA to the Δ*nat*O mutant improved UAT-B production more than twofold. Next, we generated a strain capable of producing 3-HBA endogenously by over-expressing a chorismatase gene involved in 3-HBA biosynthesis and further increased UAT-B production more than sixfold, reaching 40.0 ± 4.4 mg/L (Fig. 3). Moreover, by careful analysis of the metabolites of the Δ*nat*O and Δ*nat*J-L mutants, we proposed that NAT NRPS-PKS could incorporate 3-aminosalicylate to a low extent and would not recognize anthranilate as a starter unit (Fig. 1b). In summary, this work shows a case of targeted accumulation and significant production improvement of a medicinally interesting natural product by genetically manipulating the precursor supply in *Streptomycetes*, the talented industrial producers of bioactive molecules.

### Supplementary Information

**Additional file 1: Fig. S1.** HPLC–MS analysis of the UAT-B production level from the reported strain pRJ71+pRJ4/J1074, which was generated as the heterologous expression system for producing UAT-B and NATs (Shen et al. 2020). The mutant strain ΔnatO generated in the work was used as control. The data are displayed with the mass extraction of *m*/*z* 676.4, [M+Na]^+^ for UAT-B. **Fig. S2.** HPLC-MS analysis of the UAT-B production levels from the ΔnatO derivative strains, which individually contained the plasmids pRJ253 (A4p_nat-hyg5), pRJ255 (kasOp*_antG-natF + A4p_nat-hyg5), and pRJ256 (kasOp*_antG-antF + A4p_nat-hyg5). The empty vector pRJ5 was used as negative control. The data are displayed with the mass extraction of *m*/*z* 676.4, [M+Na]^+^ for UAT-B.

## Data Availability

Not applicable.

## Abbreviations

HPLC–MS: High-performance liquid chromatography–mass spectrum
UPLC: Ultra performance liquid chromatography
QqQ-MS: Triple quadrupole mass spectrometry
DMSO: Dimethyl sulfoxide
