# Supplementary material for: Targeted accumulation of selective anticancer depsipeptides by reconstructing the precursor supply in the neoantimycin biosynthetic pathway
Source: Bioresour Bioprocess. 2021 May 22;8(1):43. doi: 10.1186/s40643-021-00397-z (PMC10991326; doi:10.1186/s40643-021-00397-z)
Supplement: Supplementary file 1 — Additional file 1: Fig. S1. HPLC–MS analysis of the UAT-B production level from the reported strain pRJ71+pRJ4/J1074, which was generated as the heterologous expression system for producing UAT-B and NATs (Shen et al. 2020). The mutant strain ΔnatO generated in the work was used as control. The data are displayed with the mass extraction of m/z 676.4, [M+Na]+ for UAT-B. Fig. S2. HPLC-MS analysis of the UAT-B production levels from the ΔnatO derivative strains, which individually contained the plasmids pRJ253 (A4p_nat-hyg5), pRJ255 (kasOp*_antG-natF + A4p_nat-hyg5), and pRJ256 (kasOp*_antG-antF + A4p_nat-hyg5). The empty vector pRJ5 was used as negative control. The data are displayed with the mass extraction of m/z 676.4, [M+Na]+ for UAT-B. [file 40643_2021_397_MOESM1_ESM.ppt]

## Slide 1
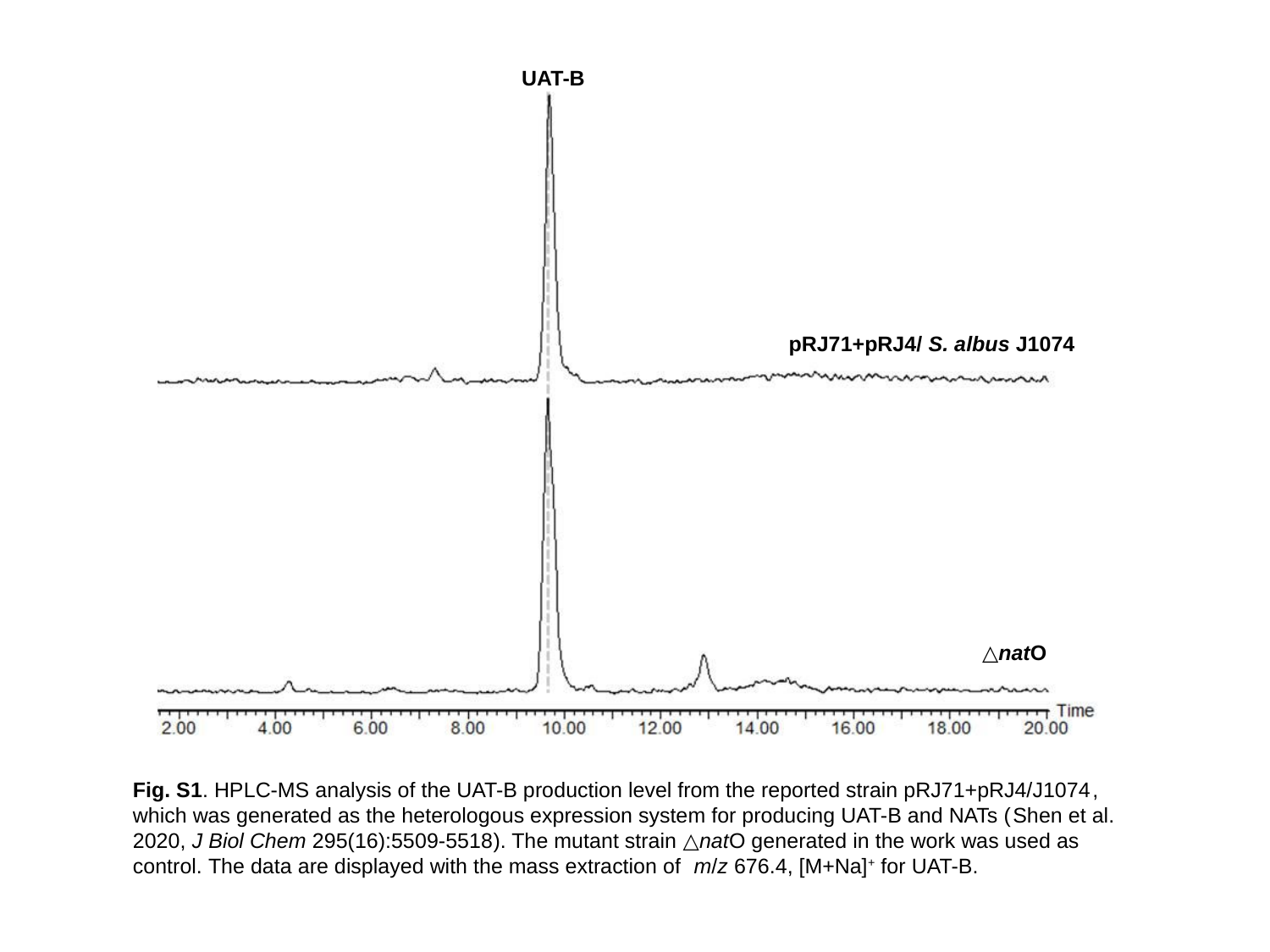

UAT-B
pRJ71+pRJ4/ S. albus J1074
△natO
Fig. S1. HPLC-MS analysis of the UAT-B production level from the reported strain pRJ71+pRJ4/J1074,
which was generated as the heterologous expression system for producing UAT-B and NATs (Shen et al.
2020, J Biol Chem 295(16):5509-5518). The mutant strain △natO generated in the work was used as
control. The data are displayed with the mass extraction of m/z 676.4, [M+Na]+ for UAT-B.

## Slide 2
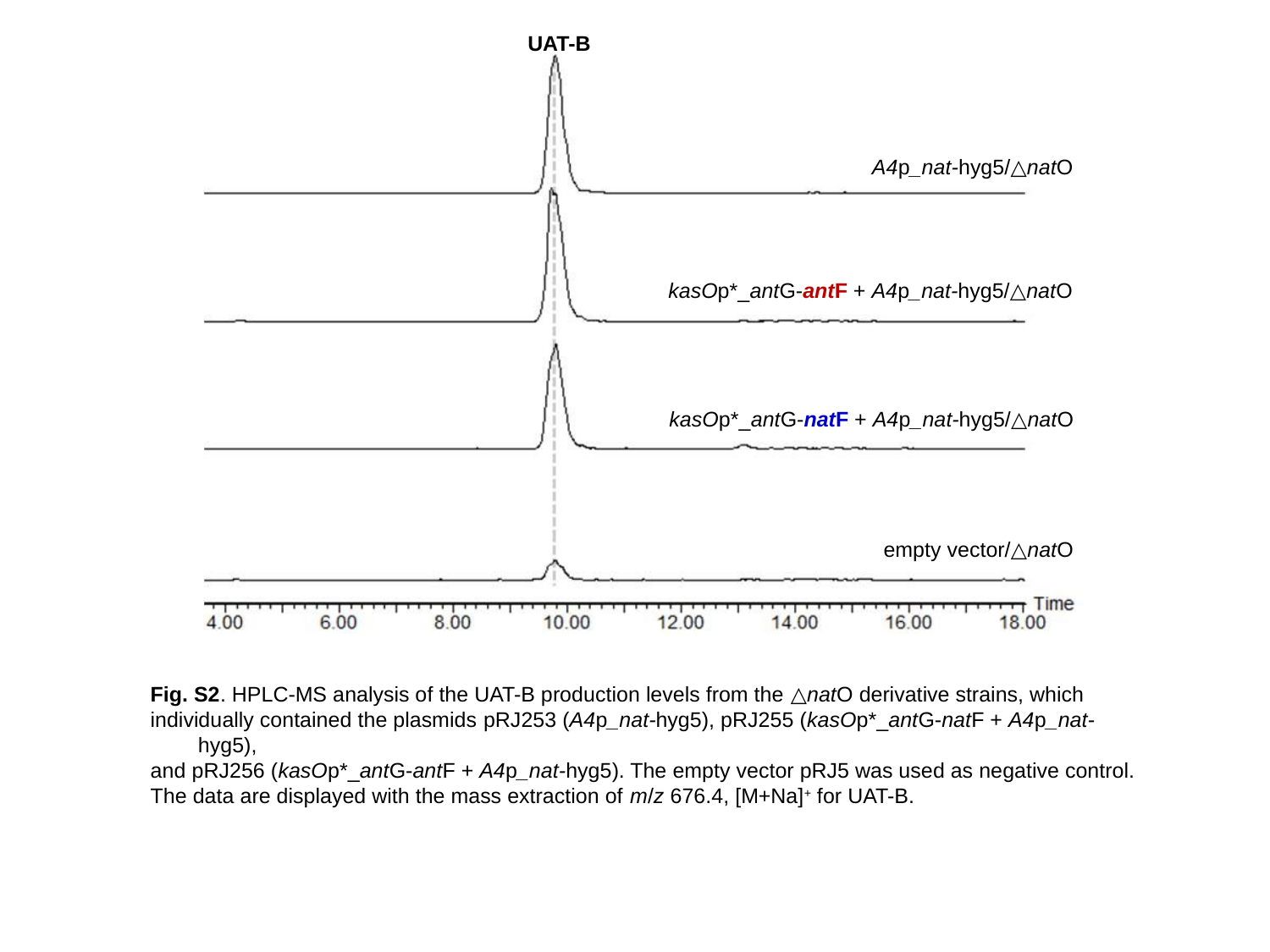

UAT-B
A4p_nat-hyg5/△natO
kasOp*_antG-antF + A4p_nat-hyg5/△natO
kasOp*_antG-natF + A4p_nat-hyg5/△natO
empty vector/△natO
Fig. S2. HPLC-MS analysis of the UAT-B production levels from the △natO derivative strains, which
individually contained the plasmids pRJ253 (A4p_nat-hyg5), pRJ255 (kasOp*_antG-natF + A4p_nat-hyg5),
and pRJ256 (kasOp*_antG-antF + A4p_nat-hyg5). The empty vector pRJ5 was used as negative control.
The data are displayed with the mass extraction of m/z 676.4, [M+Na]+ for UAT-B.
